# Effect of preheating and curing lamp distance on the degree of conversion of four nanohybrid resins: An *in vitro* study

**DOI:** 10.4317/jced.61769

**Published:** 2024-08-01

**Authors:** Loraine Uribe-Hernández, Federico Latorre-Correa, Leila Perea-Lowery, Carlos M. Ardila

**Affiliations:** 1 Prosthodontics Postgraduate Program, School of Dentistry, University of Antioquia, Medellín, Colombia; 2 Titular Professor, Prosthodontics Postgraduate Program, School of Dentistry, University of Antioquia, Medellín, Colombia; 3 Associate Professor, Institute of Dentistry, Department of Biomaterials Science, University of Turku, Turku, Finland; 4 Titular Professor, Biomedical Stomatology Research Group, School of Dentistry, University of Antioquia, Medellín, Colombia

## Abstract

**Background:**

Inadequate polymerization of resins is a major cause of failure in dental restorations. This study aimed to evaluate the hypothesis that both polymerization distance and preheating of four nanohybrid resins significantly affect their degree of conversion (DC).

**Material and Methods:**

Four A2-colored nanohybrid resins were selected: Filtek Z250 XT (3M), Tetric N-Ceram (Ivoclar), Zafira (New Stetic), and Spectra Smart (Dentsply). These resins were chosen due to their varied compositions. Forty-eight discs (6 mm diameter, 2 mm thickness) were manufactured, with 24 discs preheated to 39°C. All discs were polymerized for 40 seconds at distances of 1 mm and 6 mm using the Bluephase N lamp (Ivoclar Vivadent), operating at 385-515 nm and 1200 mW/cm². The polymerized discs were stored in distilled water at 37°C for 24 hours, and the DC was measured using Fourier-transform infrared spectroscopy (FTIR). Statistical analysis was performed using one-way ANOVA and independent samples t-tests.

**Results:**

No statistically significant differences in DC were observed between samples preheated to 39°C and those at room temperature (*p*> 0.05). Zafira exhibited the highest DC, significantly higher than Z250 XT in all groups (*p*< 0.005) and higher than Tetric N-Ceram on the surface (*p*< 0.05). Significant differences were also found between Zafira and Spectra Smart in specific conditions (*p*< 0.05). No significant differences in DC were found between polymerization distances of 1 mm and 6 mm. Uniform polymerization was achieved throughout the resin discs.

**Conclusions:**

Preheating nanohybrid resins to 39°C had no statistically significant impact on their degree of conversion. Acceptable DC values were achieved using a high-intensity lamp for 40 seconds, even at a curing distance of 6 mm. Among the tested resins, Zafira demonstrated the highest DC under various conditions, significantly outperforming Z250 XT, Tetric N-Ceram, and Spectra Smart in specific comparisons.

** Key words:**Nanohybrid composite, polymerization, degree of conversion.

## Introduction

The introduction of light-activated restorative materials has significantly impacted the field of dental restorations. Light-curing resins offer advantages such as extended working time, ease of manipulation, and improved mechanical and aesthetic properties (1). The polymerization of these resins occurs through a photoinitiator activated by light, leading to the cleavage of carbon-carbon double bonds in the monomers and the formation of new single bonds in the organic matrix of the resin, transforming monomers into polymer chains.

The polymerization process of light-curing resins is influenced by various factors including resin composition (monomers and initiators used, filler properties, resin layer thickness to be polymerized), variables related to the light source (lamp type, intensity, wavelength, light beam direction, exposure time, curing distance), and characteristics of the cavity to be restored (location, diameter, depth) (2-4). Inadequate polymerization of light-curing resins is a major cause of restoration failures (1). Monomers that do not react during polymerization may remain unreacted and later interact with environmental molecules through reactions such as oxidation and hydrolytic degradation (5). This decreases properties such as wear resistance and resin hardness, reducing the durability of the restoration (6). The degree of conversion (DC) is the parameter used to evaluate resin polymerization and provides information about the relationship between newly formed single bonds and double bonds that remain after curing (2). Fourier-transform infrared spectroscopy (FTIR) is a technique that allows quantitatively reliable analysis of the degree of conversion (7).

Previous studies have shown that the DC of composite resins typically does not exceed 80% and decreases with greater curing distances (8-13). Ideally, the lamp tip should be at 1 mm or in contact with the restoration surface (11), but this is often not feasible. Studies such as that of Hansen and Asmussen (14) described that the distance between the lamp tip and a proximal cavity of a premolar or molar ranges between 5-7 mm. Price *et al*. (15) observed that, by placing the lamp tip 3 mm away from a surface, the 10 curing lamps evaluated in their study lost less than 35% of their initial light intensity recorded at 0 mm. At 6 mm, most lamps lost more than 50% of their initial intensity. When the distance was increased to 10 mm, all lamps had lost more than 80% of their initial intensity. This suggests that the degree of conversion of resin restorations performed during clinical care will be affected, and consequently, their longevity. This is especially true for restorations in deep cavities (>4 mm) where distance, lamp tip position, and other polymerization variables are not ideal (11).

Another variable that can influence the degree of conversion is the resin temperature. In *in vitro* studies, such as those conducted by Price *et al*. (16) and AlShaafi *et al*. (17), who assessed the effect of different storage temperatures on the degree of conversion of composite resins, it was found that at higher temperatures (33°C – 35°C, respectively), the degree of conversion was higher. Reviews by Lopes *et al*. (18) and Alvarado-Santillán *et al*. (19) observed that resin preheating increases hardness and degree of conversion, reduces curing time and viscosity, allowing better polymerization and adaptation of the material to cavity walls.

The specific resins used in this study are Filtek Z250 XT (3M), Tetric N-Ceram (Ivoclar), Zafira (New Stetic), and Spectra Smart (Dentsply), chosen for their varied compositions. Filtek Z250 XT is a microhybrid composite with zirconia/silica fillers, Tetric N-Ceram contains barium glass fillers, Zafira has high-density microfillers, and Spectra Smart includes nano and microfillers.

Despite the significant insights provided by these studies, no studies were found that simultaneously considered polymerization distance and resin preheating. The frequent use of resins and the need to improve the longevity of dental restorations underline the importance of optimizing clinical protocols. This would ensure the durability of the restorations and reduce possible complications. Therefore, exhaustive studies are needed that analyze the various variables associated with the dental restoration process. These studies could help to mitigate risks and increase the success rate in dental treatments. Considering the importance of achieving optimal polymerization for the longevity and performance of dental restorations, this research aims to evaluate the effect of both curing distance and preheating on the degree of conversion of four nanohybrid resins. Therefore, two null hypotheses were proposed: 1) the DC of preheated nanohybrid resins is less than or equal to that of non-preheated resins, and 2) the DC of preheated resins polymerized at 6 mm is less than or equal to that at the shortest curing distance.

## Material and Methods

This study was approved by the Bioethics Committee of the Faculty of Dentistry at the University of Antioquia through Act No. 04 of 2022 (concept No. 109-2022).

Based on a previous study that found a DC of 58.4% and 65.1% (with an average difference of 6.7% and a standard deviation of 0.88) at different polymerization distances for nanohybrid resins (20) and considering an alpha of 0.05 and a power of 80%, a sample size of at least 9 specimens per group was determined. However, the sample comprised 48 nanohybrid resin discs with a diameter of 6 mm and a thickness of 2 mm; therefore, the sample size was increased by more than 30% to ensure greater statistical power. These A2-toned discs were manufactured using the brands Filtek Z250 XT (3M ESPE), Zafira (New Stetic), Spectra Smart (Dentsply Sirona), and Tetric N-ceram (Ivoclar Vivadent).  Table 1 provides detailed specifications of these resins according to each respective technical data sheet.

Among the commercial nanohybrid resins is Z250 3M ESPE, in which some nanoparticles are present as loosely bound nanoparticle clusters. These clusters reduce the interstitial space of the filler particles, leading to higher filler loads. The resin contains a mixture of UDMA (urethane dimethacrylate) and bis-EMA (bisphenol A diglycidyl ether dimethacrylate). These resins have a higher molecular weight, resulting in fewer double bonds per unit of weight. Additionally, their composition leads to lower polymerization shrinkage, reduced resin aging, and a lighter resin matrix. On the other hand, Tetric N-Ceram resins from Ivoclar Vivadent use a microglass filler with a particle size of 0.6 μm, providing good abrasion resistance. Spectra Smart resin from Dentsply is an aesthetic restorative material; its nanotechnology-based formula combines ideal load and weight, ensuring polishability and wear resistance. Meanwhile, Zafira resins from New Stetic have a particle size distribution ranging from 40 nm to 2.0 μm, with a total filler content by weight exceeding 78%, providing them with adequate physical, mechanical, and aesthetic properties.

**Figure 1:**
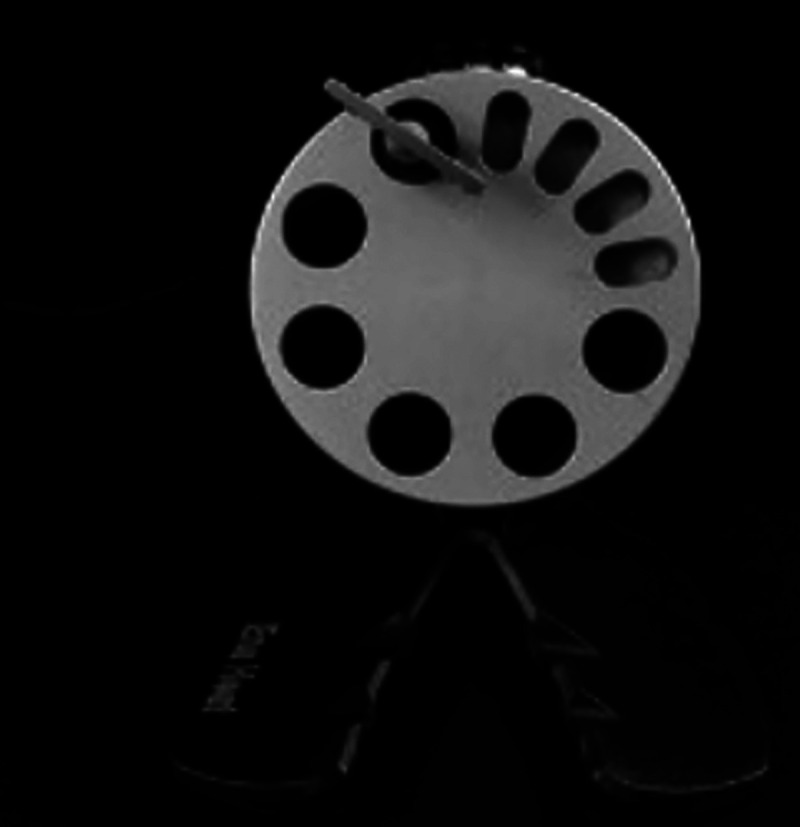
EnaHeat Heater.

The discs were divided into 4 groups, each containing 12 specimens, with 3 specimens of each resin type in each group. In groups 1 and 2, the resin was processed at a controlled temperature of 23°C using an air conditioning system and a thermohygrometer. In groups 3 and 4, the resin was preheated using the Ena Heat heater from the Micerium group (Fig. 1). This device, designed to heat resin syringes, allows reaching temperatures of 39ºC (recommended by the manufacturer for restorations) and 55ºC (recommended for resin use as a cementation material). On this occasion, the resin was preheated to a temperature of 39°C following the manufacturer’s instructions. The resins were preheated to 39°C using the Ena Heat heater from Micerium. The preheating process involved placing the resin discs in the heater for a duration of 10 minutes to ensure that they reached and stabilized at the target temperature. The temperature was continuously monitored and controlled using the built-in digital temperature display and thermostat of the Ena Heat heater, which ensures precise temperature regulation throughout the preheating period.

To avoid multiple heating cycles in samples obtained from the same resin syringe, a portion of resin with an approximate thickness of 2 mm was extracted and deposited into an empty resin syringe. Subsequently, this syringe was placed in the heater, which had been preheated and maintained for 20 minutes. The resin in the empty syringe remained in the heater for 8 minutes, as previous evaluations had determined that within this time frame, the resin portion in the syringe reached a temperature of 39°C. From this preheated resin portion, a single sample was obtained, ensuring that each resin disc received only one heating cycle.

For the creation of resin samples, a stainless-steel mold in the form of a disc with a thickness of 2 mm and a central hole of 6 mm in diameter was used. The mold was isolated with glycerin to prevent resin adhesion. The mold was placed on a Mylar sheet, and the resin was compacted into the hole using an OptraSculpt spatula (Ivoclar Vivadent) in a single 2 mm increment.

Subsequently, another Mylar sheet was placed on the upper surface of the mold, and a glass slide was placed to complete resin compaction and obtain uniform discs with the same thickness. The polymerization was then carried out for 40 seconds using a Bluephase N lamp (Ivoclar Vivadent), which had been previously calibrated with a radiometer to ensure precise parameters, such as a wavelength of 385 to 515 nm and a constant power of 1200 mW/cm², configured in high mode.

The manufacturing time for each sample was one minute, including the 40 seconds of polymerization. It is known that the resin left the heater at a temperature of 39%, but the temperature of the portion at the time of polymerization is unknown.

**Figure 2:**
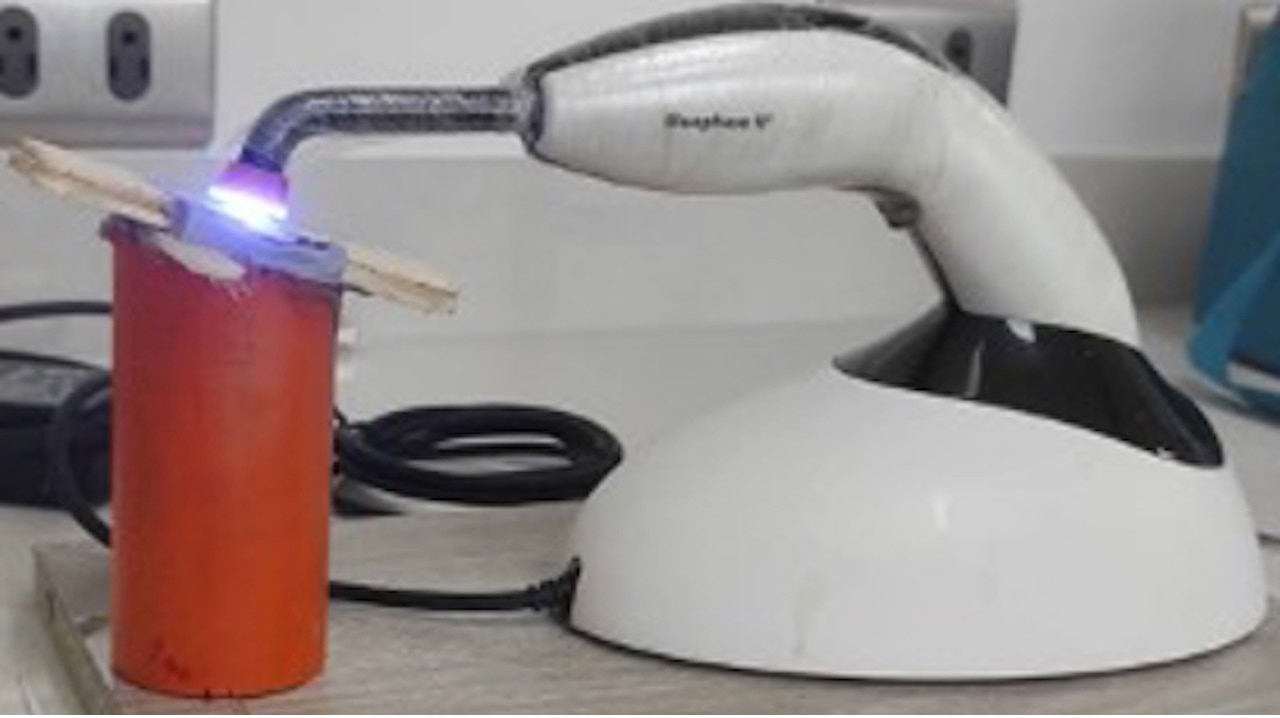
Device designed to ensure a 1 mm distance during polymerization.

The lamp tip was positioned perpendicular to the surface of the resin disc, at 1 mm in the 24 samples corresponding to groups 1 and 3, and at 6 mm in the 24 samples belonging to groups 2 and 4. To ensure this specific distance, two devices designed for this study were used (Figs. 1,2). Once the samples were removed from the mold, they were labeled and stored in distilled water inside an incubator at 37°C for 24 hours, simulating the moist conditions of the oral cavity. Subsequently, the samples were sent to the University, where they underwent Fourier-transform infrared spectroscopy (FTIR-ATR) tests to measure the degree of conversion.

Three samples from each subgroup were selected to measure the degree of conversion of the double bond. The unpolymerized material was considered as the control for each group to calculate the degree of conversion, which was analyzed using a Fourier-transform infrared spectrometer (FTIR) (Frontier FT-IR spectrometer, PerkinElmer, Llantrisant, United Kingdom). The FTIR-ATR measurements were performed with 64 scans per sample, and a spectral resolution of 4 cm^-1was utilized during the analysis.

First, the spectrum of the unpolymerized sample from each group was measured. Each polymerized specimen was ground with a file, and the obtained powder was placed on the ATR crystal for testing. The degree of conversion was measured from the aliphatic C=C peak at 1636 cm-1 and normalized in relation to the aromatic C=C peak at 1608 cm-1 using the following equation: DC%=100x(1- (Caliphatic/Caromatic)/(Ualiphatic/Uaromatic))

Where:

C is the ratio of aliphatic to aromatic peaks in the polymerized sample.

U is the ratio of aliphatic to aromatic peaks in the unpolymerized material.

Statistical Analysis. Two measurements of the degree of conversion were taken for each sample, corresponding to the surface and the bottom of each sample. These surfaces were labeled beforehand to be differentiated during the measurement.

The data of the variables included in this study (nanohybrid resin, polymerization distance, resin temperature, degree of conversion on the surface and at the bottom) were recorded in an Excel database. Subsequently, this database was exported to the statistical software IBM SPSS version 29 (IBM, United States). To establish the normal distribution of the data, the Kolmogorov-Smirnov test was used (*p* > 0.05). Descriptive statistics were employed to summarize the degree of conversion by calculating means and standard deviations.

To compare the degrees of conversion among the four resins at the same distance and temperature, as well as between the surface and the bottom of each sample, one-way ANOVA tests were conducted. In cases where the ANOVA test revealed significant differences, post-hoc multiple range analyses were performed using the Bonferroni test.

Additionally, the independent samples t-test was used to compare the degree of conversion at temperatures of 23°C and 39°C for each resin at the same distance (1 or 6 mm), both on the surface and at the bottom of each sample. Furthermore, the independent samples t-test was employed to compare the degree of conversion between distances of 1 mm and 6 mm for each resin at temperatures of 23°C or 39°C, on the surface and at the bottom of each sample.

The selection of statistical tests was based on the structure of our data and the nature of our hypotheses. Specifically: One-way ANOVA tests were utilized to compare the degrees of conversion among multiple groups (i.e., different resins) at the same distance and temperature, as well as between the surface and the bottom of each sample. Post-hoc analyses using the Bonferroni test were conducted to further examine significant differences identified by ANOVA. Independent samples t-tests were employed to compare the degree of conversion between different temperatures and distances within each resin.

## Results

The obtained values of the degree of conversion were compared both on the surface and at the bottom of each sample, and no statistically significant differences were identified (*p* > 0.05). This allows us to conclude that uniform polymerization was achieved throughout the extent of the resin discs.

**Figure 3:**
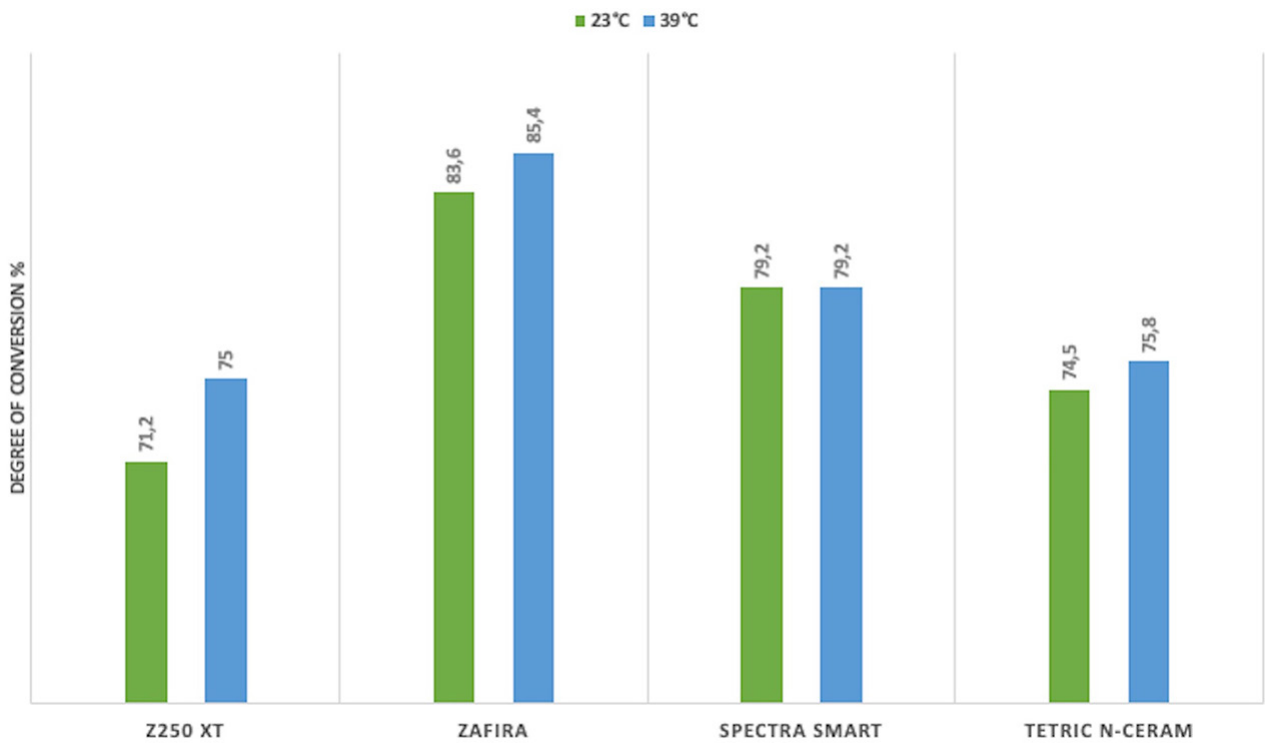
Resins polymerized at a 1 mm distance.

**Figure 4:**
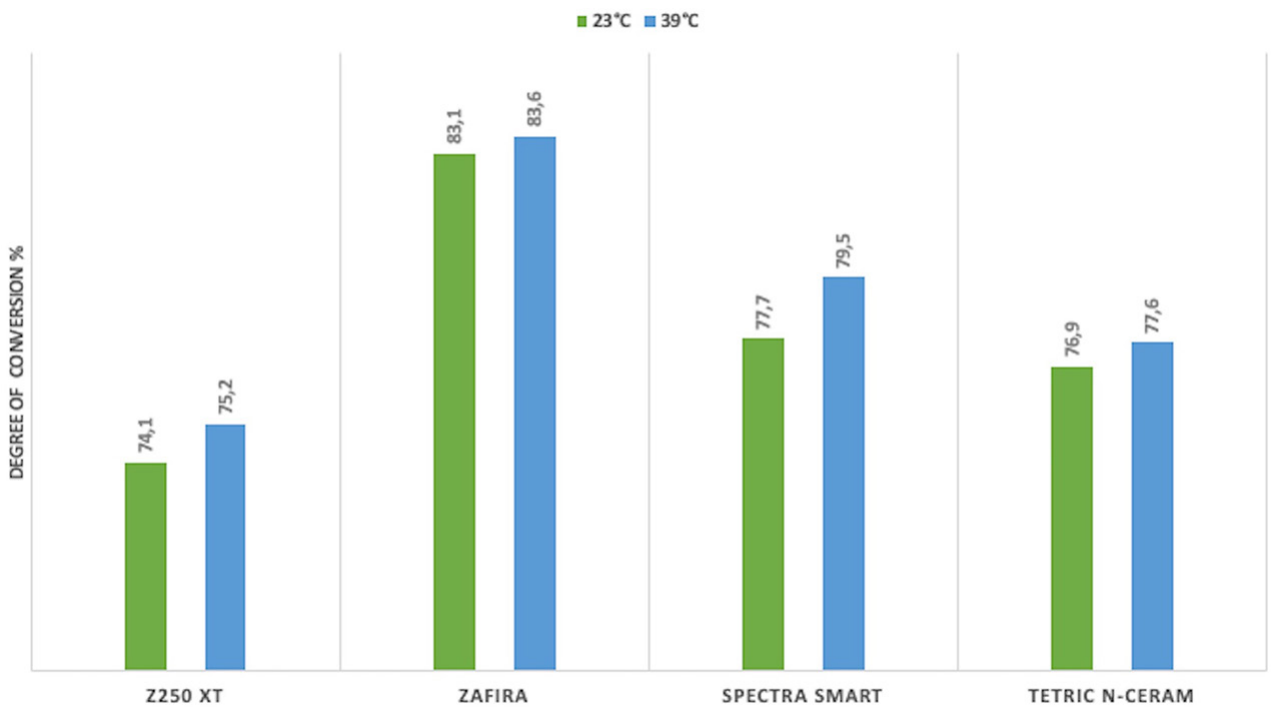
Resins polymerized at a 6 mm distance.

Among the evaluated groups, Zafira resin exhibited the highest degree of conversion, while Z250 XT resin showed the lowest degree of conversion. Statistically significant differences were found between Zafira and Z250 XT in all groups (*p* < 0.005) (Figs. 3,4), and between Zafira and Tetric N-Ceram in the values obtained on the surface of the samples (*p* < 0.05) (Figs. 3,4). Statistically significant differences were observed between Zafira and Spectra Smart in the values obtained on the surface of the samples polymerized at 6 mm distance and at a temperature of 23°C (*p* < 0.05) (Fig. 4), as well as in the samples polymerized at 1 mm distance and at a temperature of 39°C (*p* < 0.05) (Fig. 3).

Significant differences were found between Spectra Smart and Z250 XT in the samples polymerized at 1 mm and 23°C (*p* < 0.05) (Fig. 3). No statistically significant differences in the degree of conversion were found between Z250 XT and Tetric N-Ceram and between Spectra Smart and Tetric N-Ceram among the evaluated groups (*p* > 0.05) (Figs. 3,4).

Regarding the influence of resin temperature on the degree of conversion, the values obtained from the samples polymerized at the same distance but at different temperatures in the same resin were compared. No significant differences in the degree of conversion were found. However, in this study, the highest degree of conversion for each type of resin was observed in the groups where the resin was preheated: Zafira 85.4%, Spectra Smart 79.5%, Tetric N-Ceram 77.6%, and Z250 XT 75.2% (Figs. 3,4).

Additionally, to establish a relationship between the degree of conversion and the polymerization distance, the values obtained from the samples polymerized at the same temperature but at different distances in the same resin were compared. No statistically significant differences were found in this analysis.

## Discussion

The proper conversion of monomers into polymers during resin polymerization is essential for achieving restorations with satisfactory properties. The measurement of the degree of conversion can provide an estimate of the final mechanical properties of the restoration (5). Therefore, analyzing factors that can influence monomer conversion is of great importance. In this study, we evaluated whether the polymerization distance and resin temperature before polymerization affect the degree of conversion of four nanohybrid resins. The results obtained demonstrated that these two factors did not exert a significant effect on the degree of conversion, leading to the acceptance of the two null hypotheses proposed. Furthermore, no statistically significant difference was observed between the degree of conversion measured on the surface and at the bottom of each sample.

A previous study conducted by Faria-e-Silva *et al*. (21) also suggested that the distance between the lamp tip and the resin surface has a limited impact on irradiation loss and degree of conversion. This study measured the degree of conversion at various depths in 4 mm thick samples and found that the decrease in the degree of conversion was only significant at depths greater than 2 mm. Additionally, they observed that a specific resin (color A2) exhibited deeper polymerization and higher irradiation at the bottom of the samples compared to other presentations of the same resin. The findings of our study align with the observations of Faria-e-Silva *et al*. (21), as we did not find statistically significant differences in the degree of conversion between the surface and the bottom of the samples, regardless of the polymerization distance, in A2-colored resins with a thickness of 2 mm.

In another study conducted by Oh *et al*. (22), the influence of irradiation distance on the mechanical properties of different light-curing resins was evaluated. They found that irradiation distance and resin composition had a significant effect on the degree of conversion. However, by increasing the polymerization time, they managed to mitigate the effect of reduced irradiation due to increased distance, obtaining degree of conversion values with no statistically significant differences between samples polymerized at 8 mm distance for 40 seconds and samples polymerized at 0 mm distance for 20 seconds. In our study, all samples were polymerized for 40 seconds at a constant power of 1200 mW/cm², and degree of conversion values were obtained with no statistically significant differences between different polymerization distances, supporting the findings of Oh *et al*. (22).

The study conducted by Al-Zain *et al*. (23) demonstrated an inverse relationship between polymerization distance and irradiation received on the resin surface, resulting in a direct relationship between polymerization time and the distance between the lamp and the material. This allowed maintaining a similar radiant exposure but with a unique pattern for each lamp. According to their results, adequate polymerization can be achieved when radiant exposure values are between 0.7 and 1.5 J/cm², and irradiance values are between 55.9 and 84.6 mW/cm² in the deepest part of the light-exposed surface.

Patussi *et al*. (24) in a review of the available evidence on preheating and its effects on the physicochemical properties of composite resins, determined that preheating parameters (temperature, preheating time, and resin composition) are heterogeneous, making it challenging to standardize a protocol. Due to this high methodological heterogeneity, no studies evaluating the same variables or following the same parameters as this study were found, making the quantitative analysis of the results difficult.

In the study by Mundim *et al*. (20), they assessed the degree of conversion in Tetric N-Ceram resin at different temperatures (8°C, 25°C, and 60°C) using a specific heater for 30 seconds. Polymerization was performed for 20 seconds with an LED lamp at 1100 mW/cm². Unlike our study, they found statistically significant differences in the degree of conversion according to the resin temperature. The highest degree of conversion was 65.13%, corresponding to the resin preheated to 60°C, a lower value compared to our results, where Tetric N Ceram resin polymerized at 1 mm achieved 74.5% conversion at 23°C and 75.8% at 39°C, with no statistically significant differences between these two groups. The differences in results between Mundim *et al*. (20) study and our study can be associated with differences in the polymerization protocol, where our polymerization time and lamp intensity were higher.

The study conducted by Erhardt *et al*. (25) evaluated the degree of conversion of Filtek Z250 XT resin (3M ESPE) at room temperature and heated to 68°C using a specific heater for 5 minutes. It also considered the polymerization time (20-40 seconds) using an LED lamp (1300 mW/cm²) at a 1 mm distance. The results showed a degree of conversion of 68.5% in the resin polymerized for 40 seconds at room temperature and a degree of conversion of 69.8% in the resin preheated to 68°C and polymerized for 40 seconds, with no statistically significant differences in the degree of conversion. These results align with ours, where we evaluated the Z250 XT resin at temperatures of 23°C and 39°C, with no statistically significant changes in the degree of conversion.

In other studies, such as those conducted by Yang *et al*. (26) and Taubock *et al*. (27), where the degree of conversion in resins at room temperature and heated to 68°C was compared, no association was found between increased degree of conversion and increased temperature, like what we have observed in our study.

Other variables, such as the effect of preheating and cooling cycles on resins continuously shaped, have been investigated. Studies such as that by Gebril *et al*. have demonstrated that these cycles have no consequences on resin properties. However, higher temperatures could favor a better outcome, both in shrinkage and degree of conversion (30).

The study conducted by Marcondes *et al*. (30) on 10 different resins shows that composition, preheating time, and inorganic filler have a different influence on the reaction to preheating, and the viscosity of each material varies. Additionally, it is observed that temperature values decrease rapidly within an average of 10 seconds, indicating that it would not represent a clinical risk for sensitivity due to preheating and provides a guideline on heat reduction.

In summary, the results of our study are consistent with previous research that has demonstrated that preheating resins and raising the temperature to 39°C before polymerization do not have a significant effect on the degree of conversion. However, statistically significant differences were found in the degree of conversion among the different resins evaluated. Zafira resin showed a higher degree of conversion than Z250 XT and Tetric N Ceram resins. This finding could be due to the filler matrix developed for this resin, where particle sizes are modified through an industrial process called Nano Smart Position (NSP), achieving a suiTable distribution with particle sizes between 40 nm and 0.7 μm. The amount of light transmitted through resin matrix compounds is influenced by characteristics such as the size, content, microstructure, and shape of inorganic filler particles (28,29). Another factor to consider within the composition of resins and the results in degree of conversion is the photoinitiator system employed (28,29); however, further research is needed to delve into this aspect.

Among the limitations of this study is that it was conducted in a controlled environment, meaning the results obtained are valid for this specific scenario and should be interpreted with caution, as they cannot be extrapolated to the clinic. Nevertheless, this study represents a starting point for a better understanding of the relationship between the degree of conversion, polymerization protocol, and resin composition. Moreover, it is known that the resin left the heater at a temperature of 39%; however, the temperature of the portion at the time of polymerization was unknown. Clinical studies are needed to corroborate the results of this research; nevertheless, they are scarce in the literature.

Preheating resin composites to 39°C generally improves their flowability. This can be particularly advantageous in clinical settings as it allows for better adaptation of the material to the cavity walls and reduces the risk of voids. Improved flow can facilitate the placement of the resin in intricate areas of the cavity, enhancing marginal adaptation and potentially reducing microleakage.

In clinical practice, the distance between the curing light and the resin surface can vary due to the depth and location of the cavity. Our study demonstrated that a curing distance of up to 6 mm did not significantly affect the DC when using a high intensity curing lamp for 40 seconds. This suggests that clinicians can achieve adequate polymerization even in deeper or less accessible areas, if they use a sufficiently powerful curing light and adhere to recommended exposure times. Our study suggests that preheating nanohybrid resins and curing at distances up to 6 mm with high-intensity lamps do not negatively impact the DC, which is crucial for minimizing post-operative sensitivity and ensuring restoration longevity. The improved handling properties due to preheating can further enhance clinical outcomes by ensuring better adaptation and reducing shrinkage stress. These findings provide valuable insights for practitioners aiming to optimize their restorative procedures and achieve durable, patient-friendly outcomes.

## Conclusions

Considering the limitations of this study, the findings suggest that preheating the assessed resins to 39°C did not exert a statistically significant influence on the degree of conversion, although all resins exhibited a marginal elevation in their conversion values following preheating. Additionally, it was observed that with a 2 mm increase, a high-intensity lamp, and a polymerization time of 40 seconds, satisfactory DC values were achieved even at 6 mm from the resin. Significant differences were found in the DC among the different resins evaluated, with Zafira demonstrating the highest DC.

## Figures and Tables

**Table 1:** Description of the resins used in this study.

Resin	Manufacturer	Organic Matrix	Filler	Filler Weight/Volume %
Z250XT	3M ESPE, St. Paul, MN, USA	BIS-GMA, BIS-EMA, PEGDMA, UDMA and TEGDMA	Zirconium/silica modified surface of 3 microns, silica particles modified surface of 20 nm	82%/68%
Zafira	New Stetic, Medellín, Ant., Colombia	BIS-GMA, BIS-EMA, UDMA and TEGDMA	Barium glass and silicon dioxide, Ɣ-MPS fluoroboroaluminosilicate of barium, Barium borosilicate, particle size between 40 nm – 0.7µm	75-79%/55-60%
Specta Smart	Dentsply Sirona, Charlotte, NC, USA	BIS-GMA, modified with urethane, TEGDMA,	Barium aluminum borosilicate glass (BABG), silica nanoparticles, and barium aluminum fluoroborosilicate (BAGF), particle size 10 – 20 nm	75-77%/58%
Tetric N ceram	Ivoclare vivadent AG, Schaan, Liechtenstein	BIS-GMA, TEGDMA and UDMA	Barium glass, ytterbium trifluoride, silica, and mixed oxides, particle size between 40nm - 7 µm.	81%/65%

## Data Availability

The datasets used and/or analyzed during the current study are available from the corresponding author.
